# Covid and mental health in America

**DOI:** 10.1371/journal.pone.0269855

**Published:** 2022-07-22

**Authors:** David G. Blanchflower, Alex Bryson

**Affiliations:** 1 Dartmouth College, Hanover, NH, United States of America; 2 Adam Smith School of Business, University of Glasgow, Glasgow, United Kingdom; 3 NBER, Cambridge, MA, United States of America; 4 UCL Social Research Institute, University College London, London, United Kingdom; University of Luxembourg and Luxembourg Institute of Socio-Economic Research (LISER), LUXEMBOURG

## Abstract

Using 44 sweeps of the US Census Household Pulse Survey data for the period April 2020 to April 22 we track the evolution of the mental health of just over three million Americans during the COVID-19 pandemic. We find anxiety, depression and worry had two major peaks in 2020 but improved in 2021 and 2022. We show that a variable we construct based on daily inflows of COVID cases by county, aggregated up to state, is positively associated with worse mental health, having conditioned on state fixed effects and seasonality in mental health. However, the size of the effect declines in 2021 and 2022 as vaccination rates rise. For women and college educated men having a vaccine improved mental health. However, being vaccinated worsens mental health among less educated men.

## 1. Introduction

When it broke, the COVID pandemic had a sudden, unprecedented impact on the United States and its economy. Quarterly GDP in the US economy fell 1.3% in Q12020 and 8.9% in Q2. There was a marked bounce back in the third quarter of 7.5%. This of course was not unique to America as the pandemic was global just as the Great Influenza was that started during the Great War [1]. In some countries the drops in quarterly GDP (quarter on quarter) were greater: the UK saw drops of -2.5% in Q12020 and -19.4% in Q22020.

According to the U.S. Department of Labor initial weekly claims for unemployment assistance in the United States averaged just under 250,000 a week through 14^th^ March 2020 and then exploded to 2.9 million on 21^st^ March 2020 and 6 million on 28^th^ March 2020. The Department of Labor produces weekly estimates of UI claims the most recent for May 5 2022, (https://www.dol.gov/ui/data.pdf)

3/21/2020 = 2,914,107; 3/28/2020 = 5,981,787; 4/4/2020 = 6,161,308; 4/11/2020 = 4,897,867; 4/18/2020 = 4,221,556; 4/25/2020 = 3,468,261; 5/2/2020 = 2,793,245; 5/9/2020 = 2,326,632; 5/16/2020 = 2,163,595; 5/23/2020 = 1,902,793.

The official US unemployment rate reported by the Bureau of Labor Statistics (BLS) went from 3.5% in February 2020 to 4.4% in March to 14.7% in April. That turned out to be an underestimate because the systems were not set up to deal with a shock that was also a major health shock. The true rate for April 2020 was 19.7%, once account was taken of the number of workers who were classified as employed but absent from work. Special instructions were sent to household survey interviewers to ensure that all employed persons absent from work due to coronavirus-related business closures were to be classified as unemployed on temporary layoff. However, it is apparent that not all such workers were so classified. “*If the workers who were recorded as employed but absent from work due to ‘other reasons’ (over and above the number absent for other reasons in a typical April) had been classified as unemployed on temporary layoff*, *the overall unemployment rate would have been almost 5 percentage points higher than reported (on a not seasonally adjusted basis)*. *However*, *according to usual practice*, *the data from the household survey are accepted as recorded*. *To maintain data integrity*, *no ad hoc actions are taken to reclassify survey responses”*. (Employment Situation, BLS, April 2008).

**Fig 1:**
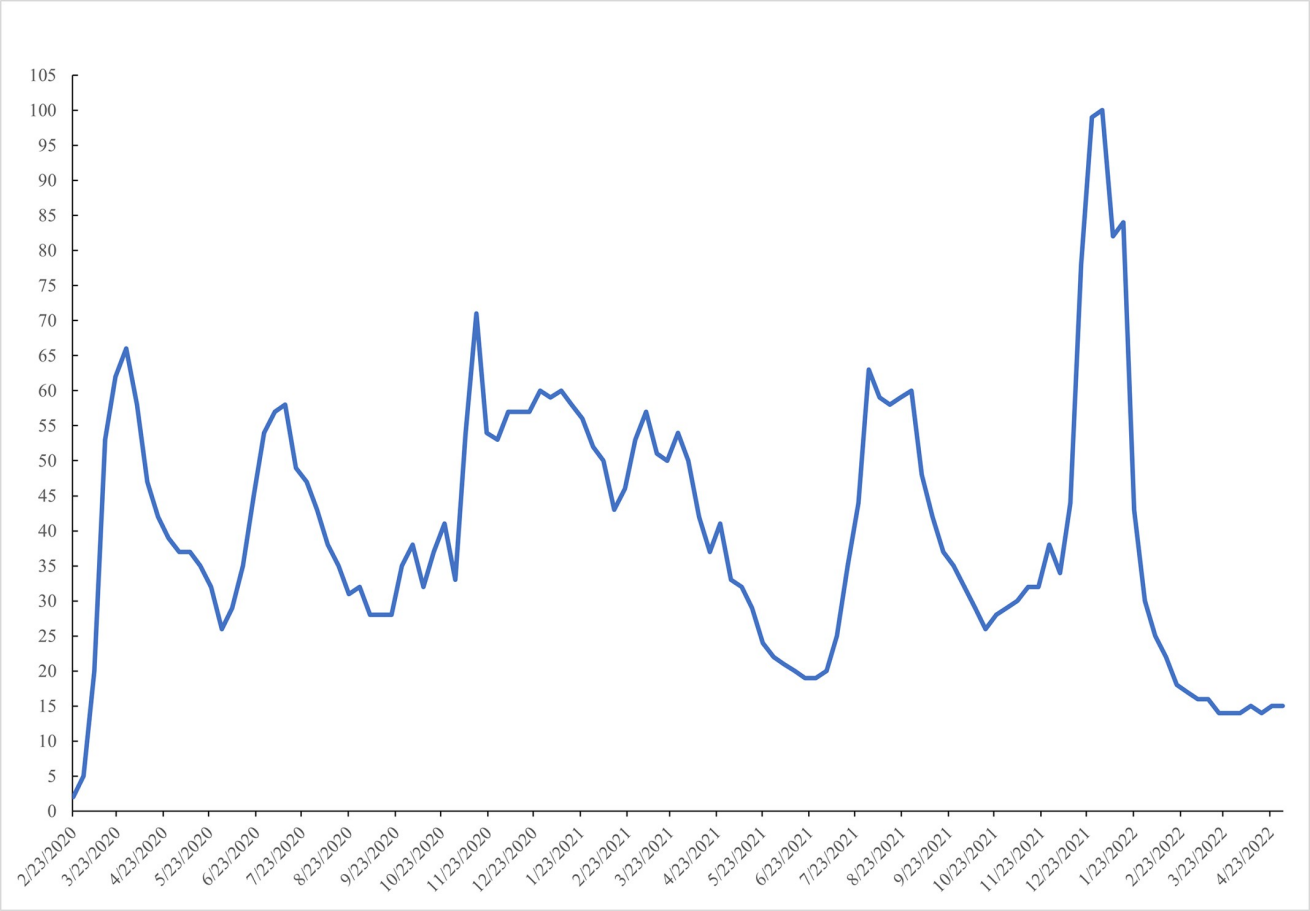
Searches for ’covid’ in google trends in the United States.

A less conventional measure of the scale of the pandemic are searches in Google Trends. Brodeur and co-authors [2] found that over the period January 1^st^, 2019, through April 10^th^, 2020, as the US went into lockdown, there was a big rise in search intensity of boredom, loneliness, worry and sadness, while there were declines in searches for stress, suicide and divorce. Fig 1 - Searches for ’Covid’ in Google Trends in the United States—plots Google Trends data showing the use of the term ‘COVID’ in the United States from March 2020-April 2022. Numbers represent search interest relative to the highest point on the figure: a value of 100 is the peak popularity for the term. A value of 50 means that the term is half as popular. A score of 0 means there was not enough data for this term. The use of the term ’covid’ jumped upwards from the start of March 2020 and there have been four subsequent peaks in July and November 2020 and August 2021 and then the highest peak at the start of 2022. We show below that this coincides with peaks in the daily in-flow rate of COVID cases.

This economic shock occurred in response to policy efforts to contain the COVID virus by going into lockdown in March 2020. On March 16, President Trump announced "*15 Days to Slow the Spread*"—a series of guidelines based on Center for Disease Control and Prevention (CDC) recommendations on topics such as physical distancing, self-isolation, and protecting those at high risk. By March 21^st^, 2020, state governors in California, New York, Connecticut and Illinois issued stay-at home orders. By the end of March many other governors had done the same. A detailed explanation by state of the dates of lockdowns is provided in Brodeur et al. (2021) [2].

The Federal government also responded in an unprecedented way with large cash transfer programs to help stimulate the economy and alleviate the financial difficulties households faced as a result of the pandemic. The largest of these is the Coronavirus Aid, Relief and Economic Security Act (CARES) costing an estimated US $2.3 trillion (or 11% of GDP) which included a US $293 billion one-time tax rebate to individuals, a $268 billion expansion of unemployment benefits, a $25 billion food safety net, a $510 billion set of loans and guarantees to prevent corporate bankruptcy, and a $349 billion small business loans and guarantees package. (See https://www.imf.org/en/Topics/imf-and-COVID-1919/Policy-Responses-to-COVID-19-19#U and https://home.treasury.gov/policy-issues/coronavirus).

The spread of disease through large pandemics, and the deaths that come with it, are often associated with fear [3] which, when compounded by restrictions on movement, social interaction and economic activity, lead to further deteriorations in mental health [4].

**Fig 2:**
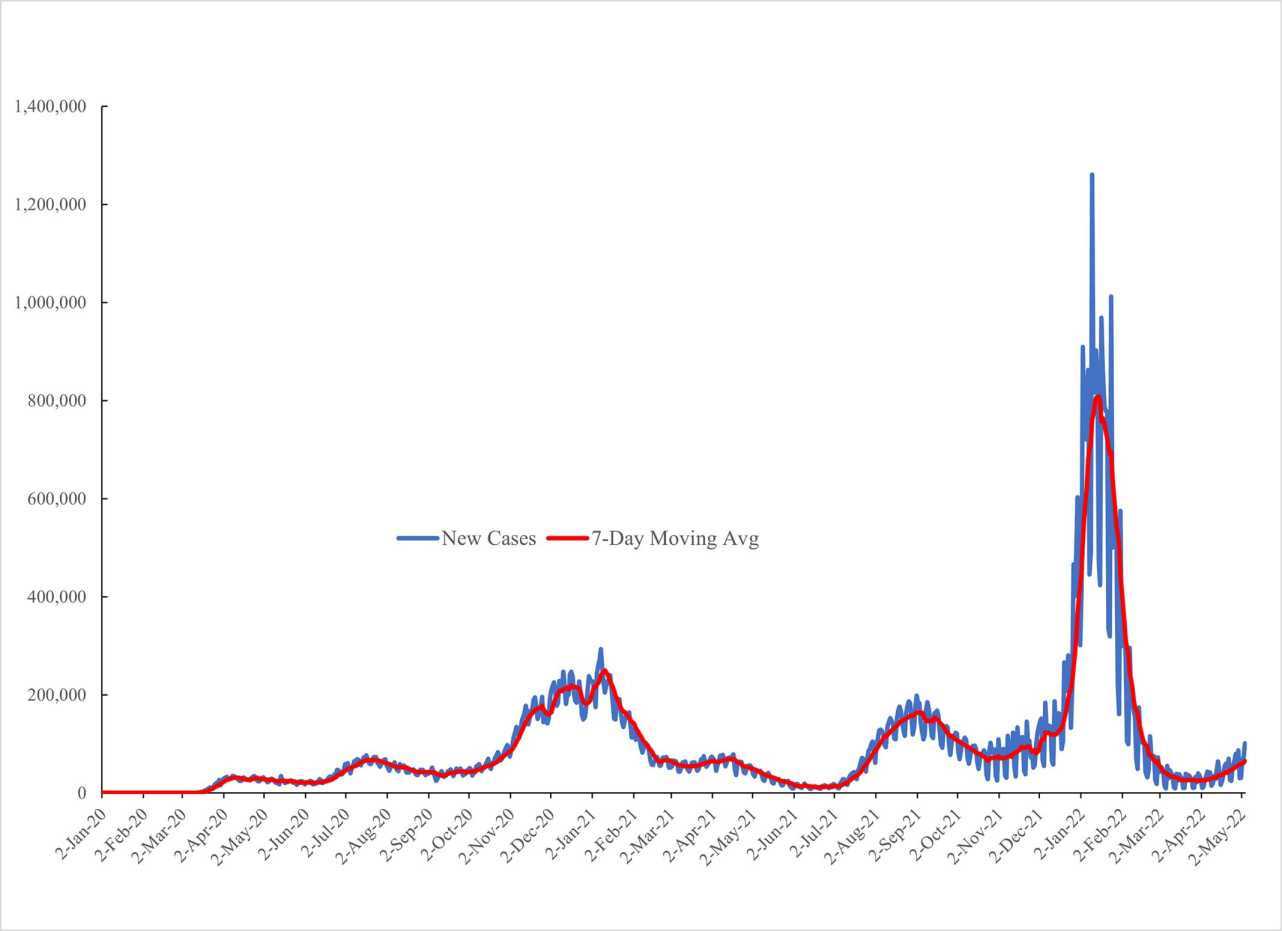
Daily cases reported to CDC 2nd January 2020-4th May 2022.

**Fig 3:**
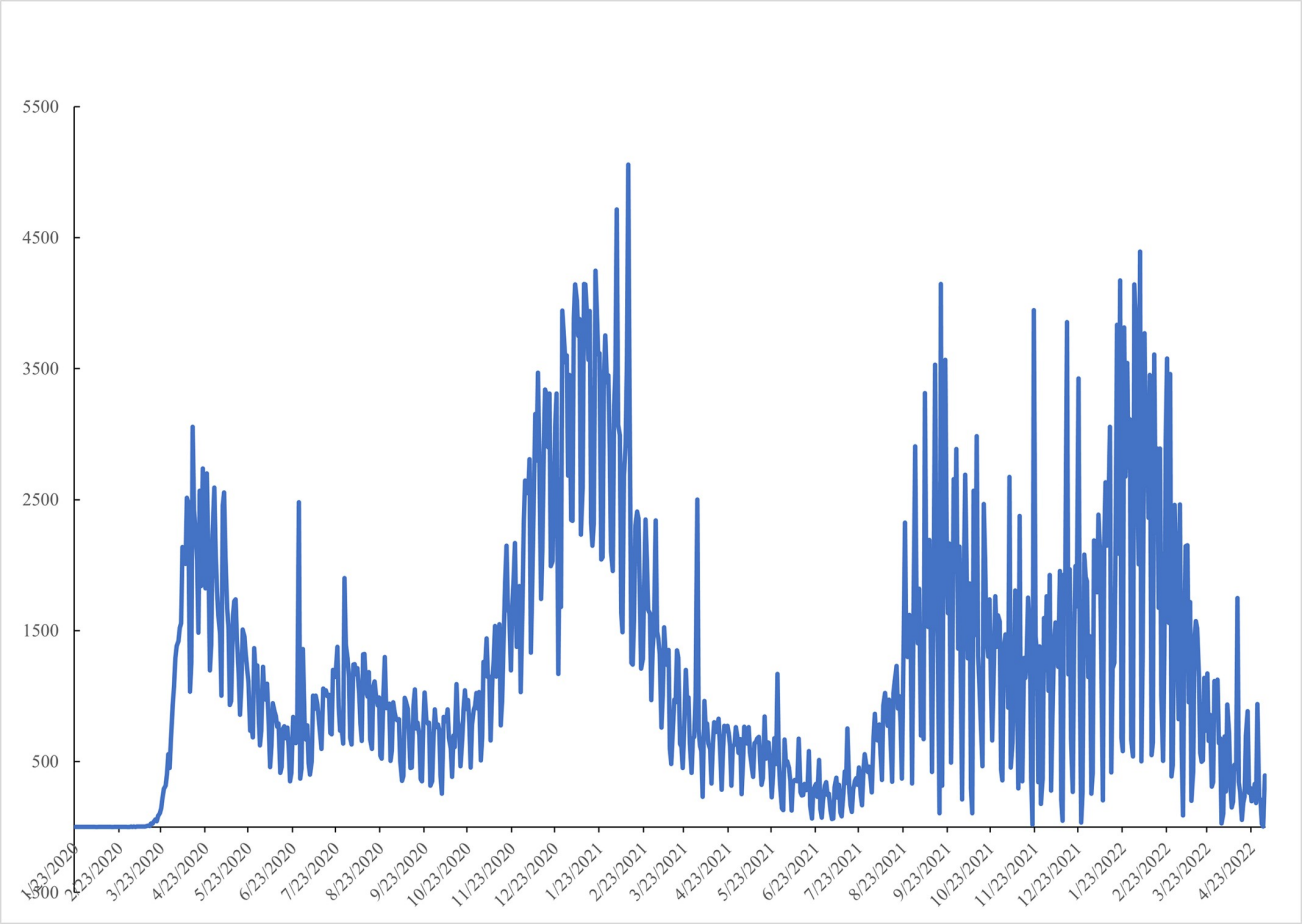
Covid daily deaths by day.

Fig 2 –Daily cases reported to CDC 2^nd^ January 2020-4^th^ May 2022—plots the daily case count of COVID-19 cases reported to the CDC as well as the seven-day moving average. There are three peaks: an early one at the start of 2021, a second one in September 2021 and the biggest one, with a peak of over a million a day, at the start of 2022. Fig 3 –Covid Daily Deaths by Day—plots daily deaths reported to the CDC which has three major peaks also, tracking the three peaks in cases shown in Fig 2 –Daily cases reported to CDC 2^nd^ January 2020-4^th^ May 2022. The first peak at the end of 2020 matches the first peak in cases in Fig 2 –Daily cases reported to CDC 2^nd^ January 2020-4^th^ May 2022 -. A second peak occurred in September 2021 and a third at the start of 2022. It is notable that the deaths in the third peak are at a comparable level to the first two peaks, conditional on a much higher case count. The death rate per case has fallen, and along with it, mental health has then been more resilient.

In this paper we contribute to the literature by analyzing high-frequency data for the United States for the period April 2020 to April 2022 to track the evolution of the mental health of nearly 3 million Americans during the COVID-19 pandemic, and its correlates. Studies for the UK indicate declining mental health during the COVID-19 crisis [5–8]. We examine the issue with a large and representative sample of Americans. Unlike most prior studies, the current one also considers the introduction and administration of the vaccines and their effects on mental health.

## 2. Mental health in the United States

**Fig 4:**
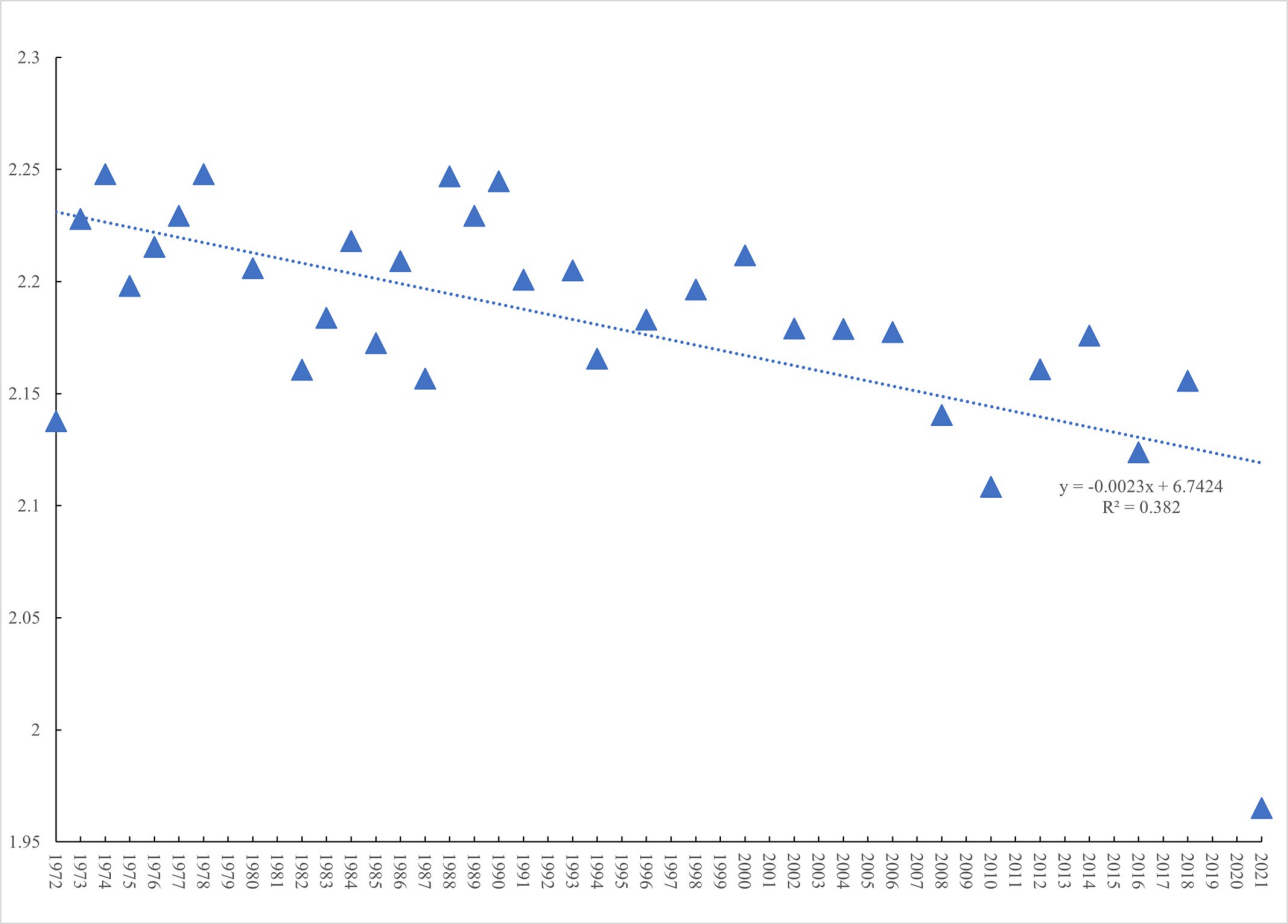
Mean happiness scores from the general social survey, 1971–2021.

Even prior to the CDC announcement of the first COVID-19 case in the United States on January 21^st^, 2020, the country had already suffered a prolonged decline in the mental well-being of its citizens. As Fig 4 –Mean happiness scores from the General Social Survey, 1971–2021—makes clear, reported happiness has been in decline since the beginning of the *General Social Survey* series in the early 1970s. This is obtained from the question ‘taken all together, how would you say things are these days—would you say that you are very happy (= 3), pretty happy (= 2), or not too happy (= 1)?’ [9].

There was a small decline during the Great Recession from 2.18 in 2008 to 2.11 in 2014, but happiness recovered quickly. In contrast, there is a very dramatic fall in happiness in 2021 to a score of 1.96 from 2.16 in 2018 the last time the survey was fielded. The proportion reporting that they were ‘very happy’, fell from 30% in 2018 to 19.5% in 2021.

Extreme distress has been steadily rising since data became available in the early 1990s [10]. Here ‘distress’ is measured with data from the Behavioral Risk Factor Surveillance System (BRFSS) (https://www.cdc.gov/brfss/index.html), using the question “*Now thinking about your mental health*, *which includes stress*, *depression*, *and problems with emotions*, *for how many days during the past 30 days was your mental health not good*?" Despair is measured as 1 if the answer is 30 last 30 days are bad mental health days. This has prompted economists to warn of a rising tide in deaths of despair [11], especially among those of prime age, the less educated, whites and natives [12]. Countries like the UK have experienced similar trends [13, 14].

To date, evidence on trends in mental health in the United States during the COVID-19 pandemic has been limited. Mental Health America (2021) [15] used a screening program to identify mental ill-health. They found that youth mental health in particular was worsening even before the COVID-19 pandemic, and the prevalence of mental illness among adults was also rising. MHA reported that 35% of the people they screened had depression while 20% had anxiety from January-September 2020. The proportion with moderate to severe anxiety rose from 71% in January to 80% in September (p. 6).

There is evidence from Gallup [16] on a small sample of 4820 adults that the percentage of people reporting 7 or higher on a life satisfaction scale (where a higher number signifies greater satisfaction) fell from 67.7% in the fall of 2019 to a low of 56.9% in April 2020. However, it subsequently rose back to 69.0% in June 2021. Analogously the percentage of people experiencing stress rose from 46% in 2019 to a peak of 60% during April 2020. The proportion of people reporting stress has now fallen back to 44% in June 2021. Worry followed a similar path.

Using a version of some of the data we use below Vahratian et al. [17] tracked the change in anxiety and depressive disorders as well as taking prescription medications or receiving counselling between August 19^th^, 2020 and February 1^st^, 2021. They found the increase in the percentage reporting symptoms on the majority of days rose most quickly among those aged under 30 (the ‘young’). They also report an increase in prescription drug taking which peaked in midlife, as found in [10]. Analysis of the KFF *Health Tracking Poll* and found that other signs of mental health deteriorated over the period including substance use, particularly among the young [18].

Adams-Prassl et al. (2022) [19] compare mental health before and after the introduction by state of lockdowns. They conducted surveys in March, April and May 2020 to examine the impact of the lockdowns and the spreading covid virus. They found that by mid-April, people’s mental health was severely affected–after the implementation of the lockdowns mental health was worse than before their introduction. They find "*the negative impact of the lockdown orders is entirely driven by a negative impact on women*". Hamermesh (2020) [20] examined data from the American Time Use Survey for 2012–2013 and used those data to simulate what would happen in lockdowns given that this would result in people staying at home. He predicted a rise in life satisfaction for married and a decline for singles due to loneliness due to more time spent alone.

The problem in the United States is to work out what was going on prior to the pandemic. The Lancet’s COVID-19 Commission on Mental Health Task Force [21] reported on the early evidence of mental health around the world in 2020 as COVID-19 hit. They reported *that "psychological distress increased during the early months of the COVID-19 pandemic and that most (but not all) facets returned to pre-pandemic levels by mid-2020*." For the United States they discussed findings from two small studies early on in the pandemic that showed sharp increases in poor mental health.

Ettman et al. found a three-fold increase in depression symptoms between 2017/18 and March-April 2020 (n = 5,065) [22]. In addition [23] reported that 13.6% of American adults indicated symptoms consistent with severe psychological distress in April 2020 (n = 1,468), an estimate nearly four times greater than was observed in 2018. Longitudinal data from the Understanding America Study was examined [24] reported a significant rise in psychological distress between March and April but a return to pre-pandemic levels by June 2020. They concluded that their data suggests that "*population level resilience in mental health may be occurring in response to the pandemic*." We also find some resiliency.

We track mental health using data on over three million people between April 2020 and April 2022. We do not have an earlier pre-pandemic baseline to compare to as the nationally representative surveys we use started in April 2020. We show that three measures of negative affect—anxiety, depression and worry and a composite measure we construct as the sum of the three–rose from April 2020 through the summer of 2020. There was a major methodological change in the survey at that point which results in a fall in all measures. However, in all four variables there is another peak around the end of 2020. The measures then decline through the middle of 2020 before picking up again mid-way through 2021. Having had covid was bad for men’s and women’s mental health. Being vaccinated was associated with better mental health among women and among educated men. However, it was associated with poorer mental health among less educated men. In what follows we also show that a variable we construct based on CDC daily inflows of covid cases by county, aggregated up to state, is positively associated with worse mental health, having conditioned on state fixed effects and seasonality in mental health, something that is sometimes overlooked in the literature.

## 3. Data and estimation

Our data are the US Census Bureau’s Public Use files of the *Household Pulse Survey (COVID-19)—*henceforth HPS—a 20-minute, online, survey conducted by the US Census Bureau gathering data for over three million people chosen randomly via residential addresses on the impact of the pandemic on their lives (https://www.census.gov/programs-surveys/household-pulse-survey.html). It began on April 23^rd^ 2020, and continues to run. The data we use in this paper end in April 2022 at sweep 44. These are repeat cross-sectional data: there is no way to link the surveys at the level of the individual respondent over time.

As reported in [17], there are methodological differences between phase 1 (April 23-July 21, 2020), phase 2 (August 19-October 26, 2020) data collection which complicate examination of trends across the two phases. These differences, they note, include a change in the data collection period from 6 days to 13 days, additional reminders sent to nonrespondents in phase 2, and elimination of a longitudinal component that was present in phase 1. Phase 3 began on October 28, 2020. Data collection for Phase 3.4 of the Household Pulse Survey started on March 2^nd^ 2022 and continued until May 9^th^, 2022. Phase 3.4 will continue with a two-weeks on, two-weeks off collection and dissemination approach. Data collection for Phase 3.3 began December 1^st^ 2021 and ended on February 7^th^ 2022. Phase 3.2 began on July 21^st^ 2021 and ended on October 11^th^ 2021. Phase 3.1 began on April 14^th^ and ended on July 5^th^, 2021. Phase 3 began on October 28^th^ 2020 and ended on March 29^th^ 2021. In 2022 the surveys become less frequent. Week #39 was September 29-October 11; week #40 December 1–13, 2021; week #41 December 29, 2021-Jan 10, 2022 (we treat this as being in 2022), week #42, January 26-February 7, 2022; week #43 March 2–14, 2022, and week #44 March 30-April 11, 2022.

We deal with this by separating out the data from phases 1, 2 and 3. The majority of the evidence we present relates to data from phase 2 and 3, both of which includes data on COVID-19 and vaccinations which are not available in phase 1.

Respondents are asked to provide information on three unhappiness measures:

*Q1*. *How often have you been feeling anxious/depressed/worried over the last seven days*?

These are based on 3 of the 4 items validated in the Patient Health Questionnaire (PHQ-4) for depression and anxiety. For details of how they were modified for the Pulse survey see Vahratian et al. (2021: 490) [17]. They are scored on an 11-step scale from 0 = "not at all" through to 11 = "completely". Although capturing slightly different aspects of ill-being, these three variables are each telling us about unhappiness. The three are highly correlated: the correlation between being anxious and worried is .81 and .70 with being down and depressed and worried and depressed have a correlation of .71. Variables like these are common in other studies. For example, [25] examined worry and depression variables in the Gallup US Daily Tracker 2008–2017 while [26] analysed worry in the Gallup World Polls of 2005–2019 and showed it was correlated with factors such as pain.

The weighted distribution of the three unhappiness measures for 2020, 2021 and for the four surveys in 2022 are presented in Table 1. They suggest that by 2022 mental health had improved on all three measures, when compared with earlier on in the pandemic. In 2020 nearly one-third (32%) of respondents said they were feeling anxious most of the time—more than half the days plus nearly every day—compared with a fifth (21%) who said they were depressed, while a quarter were worried most of the time (26%). Mental health improved in 2021, with the percent feeling bad most of the time falling on all three metrics (28% were anxious most of the time; 19% were depressed most of the time; and 22% were worried most of the time). It also seems that there is adaptation to subsequent spikes in cases, especially at the start of 2022 which saw a daily case count of over a million but a lower spike in anxiety than at earlier, lesser peaks. In Section Three we model these ordinal outcomes using OLS. Results are similar if we run ordered probits.

**Table 1:** Distribution of anxiety, depression and worry, USA 2020 and 2021 (weighted).

a) Three unhappiness measures									
	Anxious			Depressed			Worry		
	2020	2021	2022	2020	2021	2022	2020	2021	2022
Not at all	35	42	43	47	52	53	43	49	50
Several days	34	30	31	31	28	29	31	28	29
More than half the days	14	11	11	10	9	9	12	10	9
Nearly every day	18	17	15	11	11	10	14	13	12
Mean	2.14	2.01	1.98	1.86	1.79	1.76	1.95	1.86	1.84
N	1,652,390	913,308	265,772	1,651,483	912,040	265,379	1,651,189	911845	265,364
b) Unhappiness composite score (weighted)									
	2020	2021	2022						
3	29.5	36.1	37.2						
4	9.7	9.2	9.0						
5	11.2	10.3	10.7						
6	16.1	14.4	15.0						
7	6.8	6.1	5.7						
8	5.8	5.0	4.6						
9	5.9	4.9	4.5						
10	4.2	3.8	3.5						
11	3.1	2.9	2.8						
12	7.7	7.3	6.9						
Mean	5.95	5.68	5.58						
SD	2.86	2.88	2.83						
N	1,647,137	909,634	264,735						

In part b) of Table 1 we report the results from adding the three scores identified above into a composite score. Given the three scores vary between 1 and 4 the composite score varies between 3 (happiest) and 12 (least happy) with a mean of 5.57 with a sample size of just over 3 million cases.

**Fig 5:**
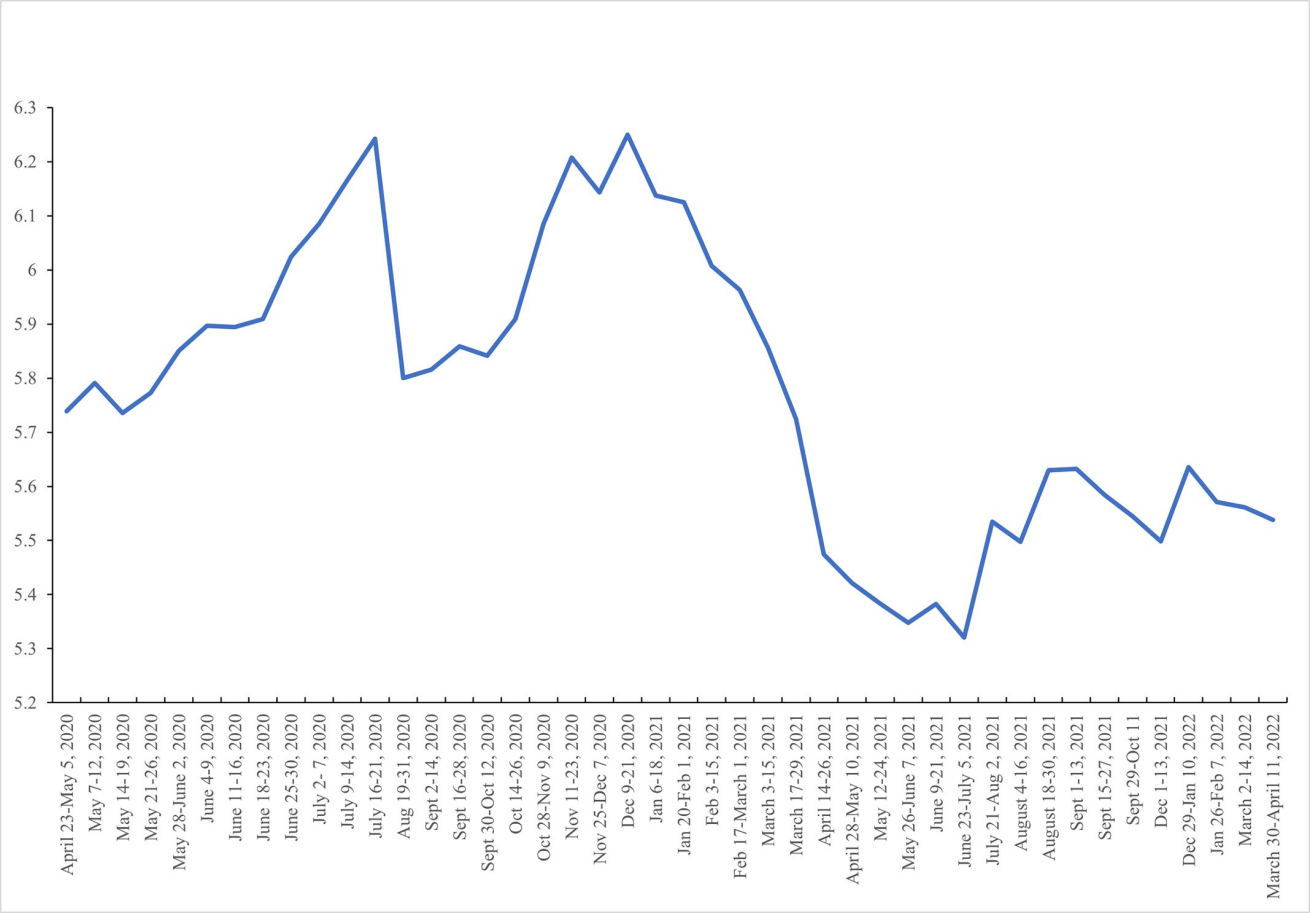
Composite unhappiness score (weighted) by week.

Fig 5 –Composite unhappiness score (weighted) by week—plots the mean composite unhappiness score by week (Mean = 5.875, n = 3,034,785). In 2020 and 2021 the gaps between weeks are mostly a single day, but the gaps are much longer in 2022. We do not plot the time path of the three individual variables anxiety, down and depressed and worried as they tracked each other, and hence the composite variable, very closely. There are two major peaks in July (6.24) and December 2020 (6.25) and a third, much lower one in September 2021 (5.63). Unhappiness was markedly lower in week 44 (5.54) than it was in week #1 in April 2020 (5.74). It is unclear how different it was to pre-pandemic levels as we have no comparable prior survey estimates to compare with.

To establish the incidence of COVID-19 infection we rely on the yes/no response to the question asked in 2021 and 2022 but not in 2020.

*Q2*. *“Has a doctor or other health care provider ever told you that you have COVID-19*?*”*

This has been asked since sweep 22 for January 6^th^-18^th^, 2021, and we have 1,596,546 respondents of whom 17.3% percent answered ‘yes’ to this question, with weights imposed. Of course, having the COVID-19 virus is a necessary but not sufficient condition to answering ‘yes’ to this question since only those who have actually been diagnosed by a doctor or health care provider will say ‘yes’. This depends, in part, on the likelihood that respondents will visit a health care provider for such a diagnosis or are sought out by health care professionals. The proportion rises by week from 14.6% in sweep 22 of the survey to 29.2% in week #44 given it is covering the integral of those who had ever had COVID-19. A big rise occurs between survey week #40 when 18.8% report having had the virus, to 29.2% in week 44.

To identify those who have received the vaccine, we use this question

*Q3*. *“Have you received a COVID-19 vaccine*?*”*

Those responding yes are coded 1 on the binary outcome identifying vaccination with a sample size of 1,612,904. Overall, 66.4% percent, weighted, of survey respondents were vaccinated when surveyed. There has been a rapid upward rise in this variable over the ten surveys that asked the question, from 7.7% in the January 6-18^th^ 2021 survey to 84.4% in week #44 in March and April 2022.

## 4. Results

We are interested in the extent to which rising covid cases, rather than lockdowns (which were triggered by the rise and an expected further surge) impacted well-being. To do so we first constructed a COVID inflow rate variable to reflect the number of cases for each state using daily CDC case data by county (https://usafacts.org/visualizations/coronavirus-covid-19-spread-map). We summed these county data to the state level and then took the average number of cases per day during the period of the survey, which are reported as cumulative case numbers. As an example, week #1 of the survey is from 23^rd^ April 2020 through 5^th^ May 2020, so we took the cumulative number of cases on May 5^th^ and deducted the number of cases from April 23^rd^ and divided by 13 as there are thirteen survey days in that sweep. The CDC also provides population counts by county, which was unchanged over the 44 weeks. We summed those to state level and divided by the population and multiplied by 1000 for simplicity. We cluster the standard errors at the week * state level giving 2244 clusters in all (51 states*44 weeks).

We should note that there are some differences in the coverage of the surveys–some cover a period of thirteen days and some six days. There are also differences in the period of time between them, until the next survey, as listed below.

Weeks 2–12 cover a six-day period with a one-day gap until the next survey

Weeks 1, 13–20, 22–26, 28–32, 34–38 cover a thirteen-day period with one-day until the next survey

Weeks 21, 27, 33, 40–42 cover a thirteen-day period with fifteen days until the next survey

Week 12 covers a six-day period with 28 days until the next survey

Week 39 covers a thirteen-day period with 50 days in between

Week 44 covers a thirteen-day period and we do not know the date of the next survey.

Data collection is currently scheduled through August 8, 2022. (https://www.census.gov/programs-surveys/household-pulse-survey/data.html#phase3.4). We treated the six-day and thirteen-day survey periods equivalently, taking the difference between the cumulative number of cases on the first and last day and dividing by six or thirteen depending on the duration of the survey, to get an average case rate by day. We then divided by the state population and multiplied by 100. This seems the appropriate way to proceed. Below we refer to the week of interview, as that is how the Census Bureau refers to each of the waves of the survey, whether they cover six or thirteen days.

Table 2 presents regressions estimating the correlates of our three mental health measures–anxiety, depression and worry—as well as the composite variable–for the full period April 2020 to April 2022. The models include our contemporaneous covid inflow rate variable, with standard errors clustered at the week*state levels. In what follows the coefficient on this covid inflow rate variable is always significantly positive in the various unhappiness equations we estimate.

**Table 2:** Regression analysis for anxiety, depression and worry and the composite unhappiness score, census household pulse surveys, April 2020—April 2022.

	Anxious	Down & Depressed	Worry	Composite
COVID-19 case rates	.4474 (6.66)	.2589 (5.32)	.3175 (5.77)	1.0246 (6.19)
Male	-.2399 (166.08)	-.1095 (89.58)	-.2174 (165.74)	-.5674 (155.44)
Age 20–24	.1510 (13.13)	.1266 (11.20)	.2156 (19.04)	.4946 (16.16)
Age 25–29	.1384 (12.26)	.0641 (5.86)	.2270 (20.52)	.4294 (14.33)
Age 30–34	.0490 (4.41)	-.0431 (4.02)	.1492 (13.62)	.1549 (5.25)
Age 35–39	-.0273 (2.45)	-.1233 (11.52)	.0795 (7.31)	-.0715 (2.43)
Age 40–44	-.0800 (7.12)	-.1539 (14.36)	.0444 (4.04)	-.1898 (6.38)
Age 45–49	-.1470 (13.07)	-.1850 (17.27)	.0080 (0.73)	-.3244 (10.86)
Age 50–54	-.2083 (18.43)	-.2129 (19.87)	-.0343 (3.09)	-.4562 (15.26)
Age 55–59	-.2989 (26.40)	-.2765 (25.54)	-.1114 (10.07)	-.6878 (22.90)
Age 60–64	-.4192 (37.17)	-.3752 (34.94)	-.2204 (19.94)	-1.0161 (34.06)
Age 65–69	-.5982 (52.42)	-.5094 (46.99)	-.3748 (33.68)	-1.4846 (49.20)
Age 70–74	-.7209 (63.90)	-.6005 (55.12)	-.4745 (42.90)	-1.7979 (59.97)
Age 75–79	-.8180 (71.77)	-.6749 (62.03)	-.5486 (49.39)	-2.0440 (67.77)
Age 80–84	-.8926 (77.68)	-.7399 (67.41)	-.6055 (53.84)	-2.2416 (73.80)
Age 85–88	-.8769 (72.72)	-.7114 (61.44)	-.5768 (49.57)	-2.1677 (68.21)
White non-Hispanic	.0166 (5.33)	.0020 (0.73)	-.0409 (14.52)	-.0219 (2.71)
Black	-.0736 (18.30)	-.0150 (4.49)	-.0020 (0.59)	-.0912 (9.02)
Asian	-.2105 (54.01)	-.0748 (22.65)	-.0864 (23.78)	-.3723 (37.26)
Other race	.1188 (27.12)	.1210 (31.81)	.1096 (27.32)	.3496 (30.98)
Government	-.2120 (83.82)	-.2549 (112.21)	-.2216 (90.72)	-.6895 (102.57)
Private company	-.2264 (124.69)	-.2453 (140.33)	-.2301 (131.44)	-.7027 (142.23)
Non-profit	-.1540 (60.58)	-.2281 (99.13)	-.1942 (80.84)	-.5773 (87.57)
Self-employed	-.1334 (50.78)	-.1974 (83.15)	-.1578 (65.39)	-.4894 (71.55)
Family business	-.1845 (33.10)	-.2270 (46.77)	-.1897 (36.27)	-.6008 (42.34)
LF status missing	-.2040 (25.72)	-.2388 (32.93)	-.1826 (25.02)	-.6260 (30.92)
Some high school	-.0547 (5.08)	-.0545 (5.20)	-.0443 (4.27)	-.1550 (5.32)
HS Diploma/GED	-.1196 (12.71)	-.1551 (16.83)	-.1389 (14.91)	-.4157 (16.10)
Some college	-.0345 (3.68)	-.1296 (14.11)	-.1124 (12.05)	-.2785 (10.79)
Associate degree	-.0826 (8.57)	-.1820 (19.58)	-.1599 (16.63)	-.4267 (16.11)
Bachelor degree	-.1402 (14.71)	-.2827 (30.53)	-.2705 (28.73)	-.6957 (26.58)
Graduate degree	-.1368 (14.46)	-.3149 (34.20)	-.2953 (31.70)	-.7492 (28.95)
2020				
May 7–12	-.0077 (0.81)	.0312 (3.64)	.0114 (1.40)	.0350 (1.43)
May 14–19	-.0043 (0.56)	.0312 (4.39)	.0084 (1.26)	.0359 (1.73)
May 21–26	-.0313 (4.10)	.0326 (4.64)	-.0019 (0.29)	-.0007 (0.04)
May 28-June	-.0175 (2.13)	.0422 (5.80)	.0125 (1.76)	.0370 (1.73)
June 4–9	.0061 (0.70)	.0471 (6.45)	.0298 (4.12)	.0832 (3.72)
June 11–16	.0460 (5.50)	.0610 (8.11)	.0691 (9.53)	.1763 (8.00)
June 18–23	.0375 (4.43)	.0583 (8.04)	.0647 (8.70)	.1611 (7.29)
June 25–30	.0688 (7.72)	.0842 (11.44)	.1020 (13.61)	.2553 (11.23)
July 2–7	.0846 (9.73)	.0932 (11.99)	.1142 (14.87)	.2924 (12.64)
July 9–14	.1086 (12.43)	.1062 (14.41)	.1366 (16.38)	.3517 (15.14)
July 16–21	.1290 (14.92)	.1232 (15.89)	.1577 (20.19)	.4109 (17.74)
Aug 19–31	.0402 (4.95)	.0640 (9.05)	.0592 (8.37)	.1636 (7.71)
Sept 2–14	.0370 (4.10)	.0645 (7.75)	.0571 (7.74)	.1592 (6.71)
Sept 16–28	.0617 (5.59)	.0875 (9.76)	.0776 (8.71)	.2267 (8.08)
Sept 30-Oct 12	.0604 (5.97)	.0759 (9.43)	.0720 (8.21)	.2089 (8.03)
Oct 14–26	.0793 (9.60)	.0947 (13.41)	.0925 (13.14)	.2672 (12.75)
Oct 28-Nov 9	.1739 (**1**6.34)	.1348 (16.28)	.1594 (16.20)	.4685 (16.97)
Nov 11–23	.1827 (**1**9.85)	.1432 (19.41)	.1631 (21.77)	.4889 (21.66)
Nov 25-Dec 7	.1224 (**1**3.74)	.1380 (18.07)	.1289 (17.27)	.3899 (17.14)
Dec 9–21	.1311 (15.24)	.1592 (20.67)	.1377 (18.17)	.4280 (18.96)
2021				
Jan 6–18	.1545 (16.19)	.1440 (18.31)	.1422 (16.81)	.4421 (17.87)
Jan 20-Feb 1	.1277 (15.40)	.1307 (17.76)	.1204 (17.01)	.3802 (17.83)
Feb 3–15	.0781 (9.82)	.1245 (17.32)	.0956 (13.22)	.2987 (14.10)
Feb 17-Mar 1	.0374 (4.02)	.1076 (14.64)	.0600 (7.65)	.2063 (8.98)
March 3–15	-.0013 (0.18)	.0788 (11.63)	.0380 (5.71)	.1149 (5.83)
March 17–29	-.0492 (6.32)	.0416 (6.02)	-.0008 (0.12)	-.0082 (0.40)
April 14–26	-.1448 (17.46)	-.0212 (2.80)	-.0801 (10.80)	-.2466 (11.10)
April 28-May 10	-.1700 (21.03)	-.0347 (4.76)	-.0901 (12.70)	-.2946 (13.77)
May 12–24	-.1868 (24.72)	-.0508 (7.20)	-.1029 (14.61)	-.3399 (16.59)
May 26-June 7	-.2066 (25.73)	-.0607 (8.77)	-.1096 (16.65)	-.3769 (18.64)
June 9–21	-.1980 (21.85)	-.0582 (7.87)	-.1016 (12.85)	-.3578 (15.37)
June 23-July 5	-.2151 (25.20)	-.0729 (10.37)	-.1150 (16.31)	-.4029 (18.89)
July 21-Aug 2	-.1612 (17.27)	-.0275 (3.60)	-.0634 (8.02)	-.2510 (10.59)
Aug 4–16	-.1337 (16.59)	-.0275 (3.78)	-.0510 (6.94)	-.2112 (9.77)
Aug 18–30	-.0883 (9.91)	.0054 (0.71)	-.0114 (1.43)	-.0937 (4.02)
Sept 1–13	-.0886 (10.02)	.0118 (1.67)	-.0145 (1.95)	-.0905 (4.11)
Sept 15–27	-.1043 (11.59)	.0041 (0.52)	-.0240 (3.11)	-.1232 (5.25)
Sept 29-Oct 11	-.1170 (13.10)	-.0023 (0.30)	-.0304 (4.04)	-.1491 (6.52)
Dec 1–13	-.1311 (12.33)	-.0014 (0.17)	-.0415 (4.40)	-.1737 (6.20)
2022				
Dec 29-Jan 10	-.1432 (10.54)	-.0068 (0.65)	-.0558 (4.97)	-.2057 (6.09)
Jan 26-Feb 7	-.1171 (11.35)	.0168 (1.98)	-.0320 (3.71)	-.1315 (5.00)
March 2–4	-.0902 (10.24)	.0086 (1.10)	-.0152 (2.04)	-.0959 (4.16)
March 30-April 11	-.1218 (14.11)	.0045 (0.59)	-.0276 (3.54)	-.1439 (6.23)
Constant	2.6086	2.3755	2.3603	7.3473
R^2^	.1000	.0714	.0859	.0999
N	3,038,753	3,036,099	3,035,423	3,028,265

Notes: Excluded reference categories: age 18–19; married; not working; less than high school, white Hispanic; April 23-May 5. Equations include a full set of state dummies. T-statistics in parentheses. Standard errors are clustered at the state*week of survey level (51*44 = 2244 clusters).

The patterns in the regressions in Table 2 are consistent for all three variables. Males are less unhappy than females, and White Hispanics are the least happy ethnic group. The separated are especially unhappy, as are those not working and those with the lowest levels of education, and the young. A prediction that single people would see bigger falls than married people in lockdowns in [20] isn’t supported in our unhappiness data. For example, married people saw a decline in our weighted composite unhappiness score from 5.56 in 2020 to 5.16 in 2022, compared with a fall from the always unhappier single people, from 6.66 to 6.41. Peak unhappiness for being anxious and worried and for the composite variable is ages 25–29 and ages 20–24 for being down and depressed.

Evidence from the Office of National Statistics in the UK also reported that the probability of being diagnosed with COVID-19 from their COVID-19 Infection Survey for September–December 2020, for example was highest for those ages 19 and 20 for both men and women and then steadily declined through life. (https://www.ons.gov.uk/peoplepopulationandcommunity/healthandsocialcare/conditionsanddiseases/adhocs/13098covid19infectionsurveypositivityratesbyagebysex).

Presumably this is not unrelated to that fact that young people’s social lives were heavily impacted, and worsened, by lockdowns. We also know that young people’s prospects of obtaining a job after college worsen in a bad economy [27].

The time path of coefficients on the week of interview variable are impacted by the change in methodology from week #13, August 19^th^-31^st^ onwards. The coefficients rise steadily from week #1 through week #12 July 16^th^- July 21^st^ and then drop sharply and then rise again reaching a maximum in November 2020 coincidentally, although it is unclear whether causally, around the date of the US presidential election, on November 3^rd^. There is evidence from previous research that the electorate tend to vote out incumbent leaders when subjective well-being is deteriorating (Ward, 2020) [28]. Of course, the time effects we are capturing will pick up many other sorts of concomitant social, political and cultural events that have happened in the US during that time which might also have had an impact on mental health, such as remote working and home-schooling during lockdowns.

We also estimated an equivalent equation for the probability of receiving prescription drugs, which is available in the data from week 13 which rose steadily from 19.4% at the start in week #1 to 23.5% at week #44. The covid flow rate variable was insignificant but there was a U-shaped pattern in age with a maximum in the forties as found in [29].

The covid flow rate variable was insignificant but there was a U-shaped pattern in age with a maximum in the forties as found, for example in [25]. The age coefficients were as follows, with 18–19 the excluded category– 20–24 = .028; 25–29 = .042; 30–34 = .046; 35–39 = .053; 40–44 = .057; 45–49 = .057; 50–54 = .054; 55–59 = .035; 60–64 = .009; 65–69 = -.027; 70–74 = -.056; 75–79 = -.083; 80–84 = -.117; 85–88 = -.135.

**Fig 6:**
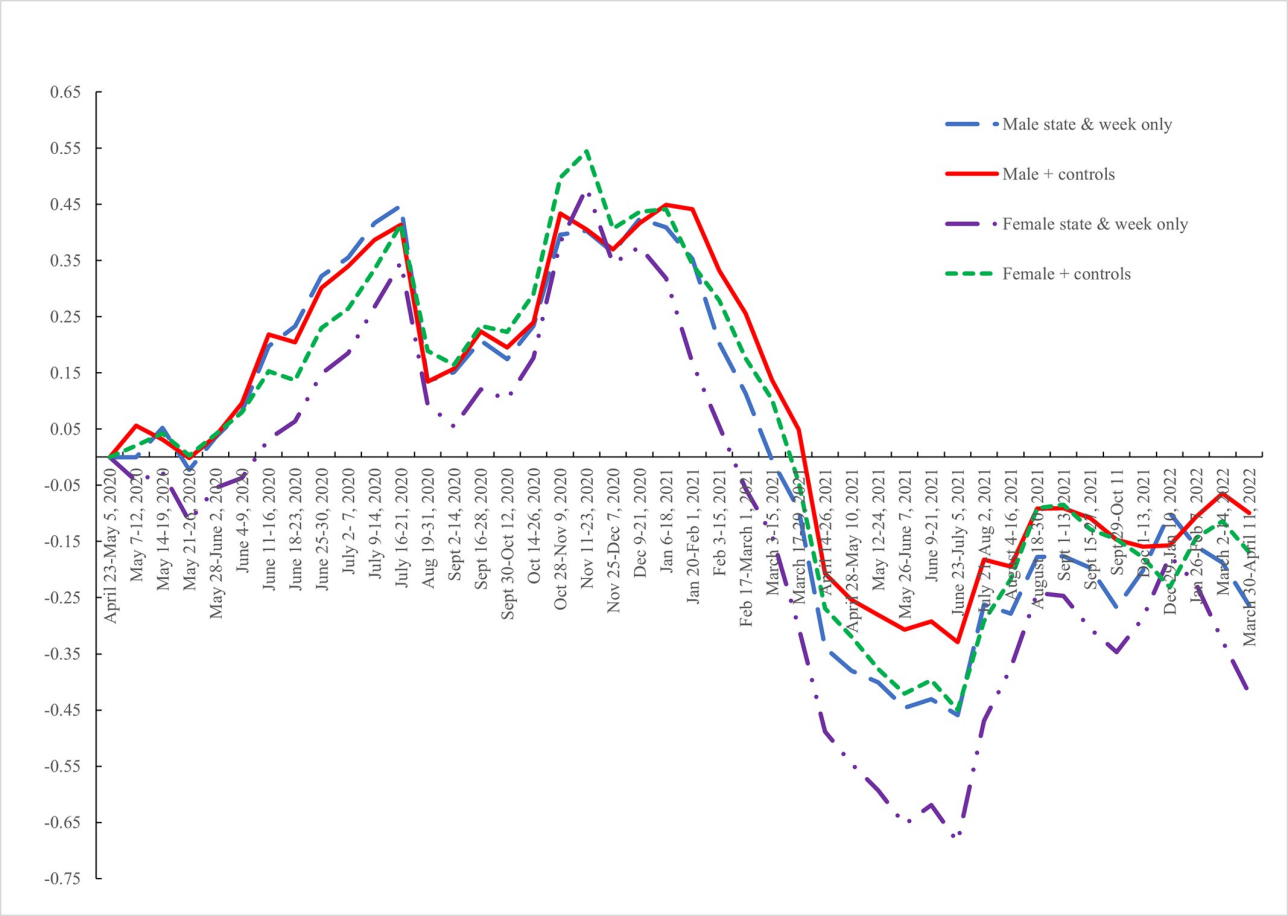
Composite scores by gender, with and without controls.

Given the evidence in earlier papers [19, 30] that women were especially impacted by this covid recession, in Table 3 we re-estimated the composite equation separately for men and women with and without the covid inflow variable. For both men and women, the covid inflow rate variable is significant and positive but with a considerably larger effect for women (+1.2) than men (+.73). Of note is that the time paths of the coefficients of the week of interview variables are largely unaffected when the covid in-flow variable is introduced (compare the first and second columns). But the patterns for men and women are different as we show in Fig 6 - Composite scores by gender, with and without controls—which plots the coefficients in column 2 for men and column 4 for women along with those from a regression excluding controls other than week of interview and state. They have similar paths, and unhappiness is lower in 2022 than in 2020 and female unhappiness dipped especially sharply at the end of 2021.

**Table 3:** Regression analysis for composite unhappiness score and time by gender, census household pulse surveys, April 2020—April 2022.

	Males		Females	
COVID-19 inflow rates		7313 (4.31)		1.2079 (6.19)
Age 20–24	.6672 (15.37)	.6674 (15.37)	.3287 (7.90)	.3292 (7.92)
Age 25–29	.8150 (19.29)	.8150 (19.30)	.1036 (2.56)	.1041 (2.57)
Age 30–34	.6774 (16.49)	.6774 (16.49)	-.2482 (6.23)	-.2476 (6.22)
Age 35–39	.4750 (11.59)	.4750 (11.59)	-.4874 (12.18)	-.4869 (12.17)
Age 40–44	.3062 (7.46)	.3062 (7.46)	-.5791 (14.40)	-.5786 (14.39)
Age 45–49	.1286 (3.13)	.1287 (3.13)	-.6910 (17.15)	-.6904 (17.14)
Age 50–54	-.0228 (0.56)	-.0227 (0.55)	-.8116 (20.11)	-.8108 (20.10)
Age 55–59	-.2495 (6.03)	-.2494 (6.03)	-1.0486 (25.91)	-1.0479 (25.90)
Age 60–64	-.5928 (14.49)	-.5927 (14.48)	-1.3693 (34.03)	-1.3686 (34.02)
Age 65–69	-1.1015 (26.83)	-1.1014 (26.82)	-1.8151 (44.80)	-1.8144 (44.80)
Age 70–74	-1.4306 (34.85)	-1.4305 (34.84)	-2.1256 (52.87)	-2.1248 (52.87)
Age 75–79	-1.6699 (40.66)	-1.6698 (40.65)	-2.3882 (58.67)	-2.3873 (58.67)
Age 80–84	-1.8370 (44.41)	-1.8370 (44.41)	-2.6301 (62.81)	-2.6296 (62.83)
Age 85–88	-1.7703 (40.93)	-1.7702 (40.92)	-2.5677 (57.59)	-2.5671 (57.58)
White non-Hispanic	-.1016 (9.43)	-.1015 (9.42)	.0301 (3.09)	.0303 (3.11)
Black	-.1101 (7.82)	-.1100 (7.81)	-.0802 (6.40)	-.0799 (6.38)
Asian	-.3687 (27.38)	-.3686 (27.38)	-.4029 (30.38)	-.4026 (30.36)
Other race	.3042 (19.16)	.3043 (19.17)	.3749 (26.81)	.3752 (26.84)
Government	-.9367 (95.30)	-.9367 (95.30)	-.5471 (67.42)	-.5471 (67.42)
Private company	-.9253 (130.29)	-.9253 (130.33)	-.5613 (92.38)	-.5613 (92.38)
Non-profit	-.6831 (61.45)	-.6831 (61.45)	-.5121 (64.29)	-.5121 (64.29)
Self-employed	-.6206 (65.89)	-.6207 (65.89)	-.4115 (45.23)	-.4115 (45.22)
Family business	-.7129 (33.22)	-.7130 (33.22)	-.5401 (28.12)	-.5400 (28.11)
LF status missing	-.6699 (15.00)	-.6704 (15.01)	-.5776 (25.69)	-.5779 (25.71)
Some high school	-.0998 (2.34)	-.0997 (2.34)	-.1997 (5.15)	-.1998 (5.15)
HS Diploma/GED	-.3208 (8.85)	-.3206 (8.84)	-.4868 (14.09)	-.4868 (14.09)
Some college	-.2272 (6.29)	-.2270 (6.29)	-.3244 (9.38)	-.3244 (9.38)
Associate degree	-.3464 (9.38)	-.3462 (9.38)	-.4961 (14.11)	-.4962 (14.11)
Bachelor degree	-.5746 (15.92)	-.5744 (15.92)	-.7911 (22.62)	-.7911 (22.62)
Graduate degree	-.6480 (17.95)	-.6478 (17.95)	-.8329 (24.08)	-.8328 (24.08)
2020				
May 7–12	.0550 (1.95)	.0561 (2.01)	.0187 (0.63)	.0206 (0.71)
May 14–19	.0300 (1.33)	.0314 (1.41)	.0400 (1.49)	.0422 (1.63)
May 21–26	-.0037 (0.17)	-.0019 (0.09)	.0003 (0.01)	.0032 (0.13)
May 28-June	.0361 (1.63)	.0378 (1.73)	.0381 (1.40)	.0409 (1.55)
June 4–9	.0943 (4.38)	.0960 (4.52)	.0770 (2.54)	.0797 (2.70)
June 11–16	.2169 (8.41)	.2182 (8.55)	.1508 (5.25)	.1529 (5.47)
June 18–23	.2045 (8.91)	.2044 (9.07)	.1372 (4.83)	.1371 (4.99)
June 25–30	.3032 (13.02)	.3012 (13.14)	.2332 (7.59)	.2300 (7.82)
July 2–7	.3427 (14.35)	.3396 (14.62)	.2697 (8.84)	.2648 (9.07)
July 9–14	.3918 (14.98)	.3868 (15.29)	.3426 (11.70)	.3345 (12.07)
July 16–21	.4194 (16.83)	.4139 (17.11)	.4235 (14.01)	.4146 (14.35)
Aug 19–31	.1373 (5.64)	.1346 (5.59)	.1934 (7.36)	.1889 (7.38)
Sept 2–14	.1595 (5.85)	.1575 (5.80)	.1683 (6.23)	.1648 (6.24)
Sept 16–28	.2277 (7.20)	.2238 (7.06)	.2400 (7.64)	.2334 (7.51)
Sept 30-Oct 12	.2009 (7.20)	.1951 (6.97)	.2329 (7.61)	.2229 (7.35)
Oct 14–26	.2501 (10.91)	.2399 (10.36)	.3080 (11.01)	.2906 (10.64)
Oct 28-Nov 9	.4547 (14.31)	.4339 (13.35)	.5325 (16.32)	.4973 (15.24)
Nov 11–23	.4395 (18.54)	.4053 (16.96)	.6027 (21.52)	.5446 (19.44)
Nov 25-Dec 7	.4046 (18.25)	.3699 (15.78)	.4648 (15.96)	.4069 (13.76)
Dec 9–21	.4528 (19.42)	.4168 (17.09)	.4963 (18.46)	.4368 (15.43)
2021				
Jan 6–18	.4848 (16.50)	.4489 (14.98)	.5006 (17.96)	.4411 (15.32)
Jan 20-Feb 1	.4633 (20.26)	.4411 (18.70)	.3801 (14.30)	.3432 (12.95)
Feb 3–15	.3439 (15.64)	.3321 (15.30)	.2984 (10.62)	.2788 (10.06)
Feb 17-Mar 1	.2626 (10.37)	.2559 (10.21)	.1870 (6.50)	.1759 (6.26)
March 3–15	.1423 (6.18)	.1373 (6.03)	.1112 (4.30)	.1030 (4.10)
March 17–29	.0553 (2.43)	.0493 (2.20)	-.0347 (1.23)	-.0445 (1.59)
April 14–26	-.2003 (7.89)	-.2070 (8.26)	-.2578 (8.63)	-.2690 (9.36)
April 28-May 10	-.2508 (10.97)	-.2539 (11.24)	-.3136 (11.02)	-.3189 (11.47
May 12–24	-.2803 (11.78)	-.2804 (11.84)	-.3764 (14.49)	-.3767 (14.94)
May 26-June 7	-.3093 (13.35)	-.3070 (13.50)	-.4245 (15.56)	-.4208 (15.77)
June 9–21	-.2957 (12.48)	-.2925 (12.48)	-.4024 (13.24)	-.3972 (13.33)
June 23-July 5	-.3317 (12.90)	-.3289 (12.88)	-.4542 (17.61)	-.4496 (17.92)
July 21-Aug 2	-.1747 (6.11)	-.1822 (6.41)	-.2792 (9.69)	-.2917 (10.39)
Aug 4–16	-.1768 (7.39)	-.1943 (8.08)	-.1880 (6.84)	-.2171 (8.16)
Aug 18–30	-.0679 (2.64)	-.0913 (3.54)	-.0514 (1.81)	-.0909 (3.23)
Sept 1–13	-.0667 (2.82)	-.0909 (3.82)	-.0442 (1.52)	-.0850 (3.02)
Sept 15–27	-.0877 (3.21)	-.1087 (4.04)	-.0928 (3.09)	-.1283 (4.38)
Sept 29-Oct 11	-.1316 (4.88)	-.1472 (5.56)	-.1198 (4.16)	-.1459 (5.22)
Dec 1–13	-.1390 (5.06)	-.1593 (5.86)	-.1445 (4.08)	-.1784 (5.18)
2022				
Dec 29-Jan 10	-.0404 (1.74)	-.1569 (4.62)	-.0405 (1.40)	-.2317 (5.61)
Jan 26-Feb 7	-.0458 (1.93)	-.1061 (3.95)	-.0440 (1.56)	-.1436 (4.49)
March 2–4	-.0620 (2.54)	-.0645 (2.67)	-.1094 (3.76)	-.1136 (4.01)
March 30-April 11	-.0988 (4.34)	-.0995 (4.42)	-.1683 (5.50)	-.1694 (5.63)
Constant	6.3931	6.3828	7.7282	7.7110
R^2^	.0957	.0957	.0792	.0792
N	1,233,120	1,233,120	1,795,139	1,795,139

Notes: excluded reference categories: age 18–19; not working; less than high school, white Hispanic; April 23-May 5. Equations include a full set of state dummies. T-statistics in parentheses. Standard errors are clustered at the state*week of survey level (51*44 = 2244 clusters).

This is also consistent with earlier findings of [31], that economic activity in the USA was strongly tied to local virus conditions during the first six to nine months of the pandemic, then decoupled in late 2020 through the first half of 2021. This link, they found, strengthened again in the third quarter of 2021, particularly for highly vaccinated counties. One possible interpretation of this restrengthening, they suggest, is that areas with high vaccination rates have heightened virus risk aversion and hence high sensitivity to changes in local virus conditions.

Table 4 re-estimates the four unhappiness equations from Table 2 that covered 2020–2022 but this time dropping 2020 so as to concentrate on the data which contain vaccine status and having had COVID-19 (which are available in weeks 22–44 of the HPS). We include as controls whether the respondent reported they had been told they had COVID-19 and whether they received a vaccine and the covid inflow rate separately by gender. We include the same controls as in Table 2, but for simplicity their coefficients are not reported. In all eight instances, for both men and women the COVID-19 inflow variable by week and year is significantly positive.

**Table 4:** Regression analysis for four unhappiness variables incorporating COVID-19 inflow rates, having had COVID-19 and/or being vaccinated Census Household Pulse Surveys, 2021–2022, survey weeks 22–44.

Anxious		
	Male	Female
COVID-19 inflow rates	+.2763 (4.35)	+.3329 (4.67)
Vaccinated	+.0197 (3.46)	.0025 (0.55)
Had COVID-19	+.0101 (2.40)	+.0271 (7.57)
Constant	2.4837	3.0431
R^2^	.0936	.0714
N	559,750	814,490
b) Down & depressed		
	Male	Female
COVID-19 inflow rates	+.1178 (2.08)	+.1944 (3.71)
Vaccinated	-.0048 (1.04)	-.0401 (10.46)
Had COVID-19	.0045 (1.26)	+.0184 (5.92)
Constant	2.2608	2.7154
R^2^	.0831	.0648
N	559,016	813,513
c) Worried		
	Male	Female
COVID-19 inflow rates	+.1533 (2.86)	+.2526 (4.20)
Vaccinated	-.0021 (0.44)	-.0174 (4.38)
Had COVID-19	+.0228 (6.15)	+.0386 (11.32)
Constant	2.2223	2.7444
R^2^	.0769	.0743
N	558,899	813,307
d) Composite		
	Male	Female
COVID-19 inflow rates	+.5514 (3.40)	+.7785 (4.51)
Vaccinated	.0132 (0.44)	-.0544 (4.66)
Had COVID-19	+.0228 (6.15)	+.0843 (9.07)
Constant	6.9696	8.5157
R^2^	.0983	.0873
N	557,599	811,464

Notes: Equations include a full set of state and week dummies, plus controls for age, race, education and labor force status. T-statistics in parentheses. Standard errors are clustered at the state*week of survey level (51*23 = 1173 clusters).

For all four variables women’s unhappiness is higher where they have had COVID-19. In three of models—with anxiety being the exception—being vaccinated is significantly negative for females. For men, having had COVID-19 is significantly positive in three models—with down and depressed, being the exception. What stands out is that being vaccinated is statistically positive for men in the case of anxiety and insignificant in the remaining three variables.

One explanation for the correlation between vaccination and greater unhappiness among men could be the social stigma attached to being vaccinated. Fox News is well known to have insisted that all its staff are vaccinated and boosted despite its public opposition to vaccines—as shown in Samira Sadeque, ’Nearly all Fox staffers vaccinated for COVID even as hosts cast doubt on vaccine,’ *The Guardian*, 15^th^ September 2021 and Emma Goldberg, ’Fox tightens its vaccine rule, removing a test-out option for N.Y.C. office workers’, *New York Times*, 20^th^ December 2021.

If so, this might be stronger among the less educated. In Table 5 we report the results of estimating an equation with the composite variable as the dependent variable by gender and level of education. We report results for the less educated and the more educated, which includes respondents with at least an associate’s, bachelor’s or graduate degree. In all four cases the COVID-19 inflow rates and the individual COVID-19 variables are significant and positive. Now the vaccination variable is significantly negative in both female equations and the male equation 2, for the more educated. It is significantly positive for less educated men.

**Table 5:** Regression analysis for composite unhappiness scores by education levels, COVID-19 and vaccinations, 2021 & 2022.

	Male		Female	
	Less educated	More educated	Less educated	More educated
COVID-19 inflow rates	+.3419 (1.20)	+.6729 (3.50)	+.5169 (1.82)	+.9377 (4.69)
Vaccinated	+.0823 (4.07)	-.0366 (2.25)	-.0381 (2.38)	-.0621 (4.42)
Had COVID-19	+.0701 (3.66)	+.0335 (2.67)	+.0689 (4.49)	+.1144 (10.23)
Constant	6.7859	7.4658	8.1989	7.8571
Adjusted R^2^	.1008	.0838	.1001	0691
N	176,535	381,064	276,549	534,915

Equations include a full set of state and week dummies, plus controls for age, race, education and labor force status. T-statistics in parentheses. Standard errors are clustered at the state*week of survey level (51*23 = 1173 clusters)

It seems appropriate to explore why there has not been a spike in the unhappiness data especially at the start of 2022 when COVID-19 cases spiked, as shown in Fig 2 –Daily cases reported to CDC 2^nd^ January 2020-4^th^ May 2022, even though we found evidence that the COVID-19 inflow rate has a positive effect on our unhappiness variables. One possibility is that people have adapted to COVID-19, another is that they perceive the risk of death to be lower due to the vaccines. In Table 6 we explore this issue by first splitting the sample in half with weeks 1–22 (Jan 6–18, 2021) and then weeks 23–44. We find that the coefficient on the COVID-19 inflow variable in the first column is 2.4 versus 0.7 in the second. We also produce estimates by year which decline from 2.57 in 2020 to 1.7 in 2021 to 0.37 in 2022. All are statistically significant, but the trend is clearly declining.

**Table 6:** Regression analysis for composite unhappiness scores with sample splits, weeks 1–44.

	Weeks 1–22	Weeks 23–44	2020	2021	2022
COVID-19 inflow	2.4014 (7.83)	.7184 (5.09)	2.5665 (8.08)	1.7131 (6.25)	.3689 (3.03)
Constant	6.8763	7.4658	6.8676	7.8752	7.001
Adjusted R^2^	.0788	.0949	.0780	.0944	.0956
N	1,701,674	1,326,585	1,645,389	907,773	263,753

Equations include a full set of state and week dummies, plus controls for age, race, education and labor force status. T-statistics in parentheses. Standard errors are clustered at the state*week of survey level

In Table 7 we now move on to examining the characteristics of those who were diagnosed as having had COVID-19 using OLS, overall and for men and women separately. Once again, we include our COVID-19 inflow rate variable, which as expected comes in significantly positive, on the assumption that people may be driven or encouraged to get vaccinated when COVID-19-19 outbreaks are particularly bad. We also include a variable identifying being vaccinated which is negatively associated with being diagnosed with COVID-19-19, as expected given that the vaccines are a barrier to catching the virus. We should note that the probability of being vaccinated rose approximately linearly with age. Weighted average vaccination rates in the HPS were as follows; age 18–19 = 54.5 (80.0); 20–24 = 55.2 (70.9); 25–29 = 57.7 (79.7); 30–34 = 58.4 (77.2); 35–39 = 59.7 (79.3); 40–44 = 61.0 (80.6); 45–49 = 63.6 (82.8); 50–54 = 66.7 (85.2); 55–59 = 69.1(87.6); 60–64 = 71.7 (90.2); 65–69 = 78.7 (92.5); 70–74 = 81.5 (94.4); 80–84 = 84.2 (95.9); 85–89 = 69.8 (80.5) with 2022 rates in parentheses.

**Table 7:** Regression analysis for diagnosed with COVID-19, 2021 & 2022.

	All	Male	Female
COVID-19 inflow rates	.1140 (4.76)	.1391 (4.89)	.0965 (3.38)
Vaccinated	-.0823 (43.22)	-.0837 (34.47)	-.0813 (43.79)
Male	-.0132 (20.49)		
Age 20–24	.0336 (6.62)	.0303 (4.29)	.0349 (5.06)
Age 25–29	.0278 (5.61)	.0274 (3.98)	.0256 (3.94)
Age 30–34	.0177 (3.71)	.0166 (2.53)	.0156 (2.47)
Age 35–39	.0143 (2.96)	.0132 (2.03)	.0123 (1.93)
Age 40–44	.0185 (3.91)	.0189 (2.89)	.0155 (2.47)
Age 45–49	.0223 (4.72)	.0233 (3.55)	.0191 (3.05)
Age 50–54	.0166 (3.57)	.0247 (3.86)	.0090 (1.44)
Age 55–59	.0055 (1.17)	.0169 (2.62)	-.0043 (0.70)
Age 60–64	-.0139 (2.97)	.0013 (0.21)	-.0265 (4.26)
Age 65–69	-.0325 (6.87)	-.0141 (2.19)	-.0475 (7.57)
Age 70–74	-.0420 (8.72)	-.0254 (3.89)	-.0566 (8.86)
Age 75–79	-.0472 (9.56)	-.0298 (4.48)	-.0636 (9.79)
Age 80–84	-.0526 (10.40)	-.0336 (4.98)	-.0715 (10.79)
Age 85–89	-.0464 (8.92)	-.0300 (4.26)	-.0646 (9.16)
White non-Hispanic	-.0614 (30.19)	-.0569 (23.37)	-.0634 (26.83)
Black	-.0499 (21.61)	-.0464 (15.77)	-.0521 (19.21)
Asian	-.0828 (36.22)	-.0760 (27.85)	-.0868 (30.49)
Other races	-.0397 (16.05)	-.0357 (11.83)	-.0420 (13.52)
Government	.0209 (16.52)	.0240 (12.93)	.0197 (12.91)
Private company	.0151 (16.47)	.0146 (11.08)	.0165 (14.86)
Non-profit	.0197 (14.93)	.0177 (8.54)	.0199 (12.98)
Self-employed	.0036 (3.16)	.0128 (7.90)	-.0047 (3.08)
Family business	.0082 (2.95)	.0147 (3.39)	.0044 (1.20)
LF Status missing	.0326 (7.43)	.0298 (3.45)	.0330 (6.48)
Some high school	-.0275 (5.48)	-.0380 (5.19)	-.0205 (2.99)
High school graduate	-.0327 (7.15)	-.0409 (6.26)	-.0260 (4.24)
Some college	-.0315 (6.80)	-.0380 (5.80)	-.0261 (4.29)
Associate degree	-.0254 (5.37)	-.0353 (5.27)	-.0189 (3.05)
Bachelor degree	-.0507 (10.59)	-.0518 (7.76)	-.0496 (7.91)
Graduate degree	-.0638 (13.28)	-.0644 (9.67)	-.0636 (10.08)
2021			
Jan 20-Feb 1	.0064 (2.73)	.0033 (1.03)	.0083 (3.52)
Feb 3–15	.0169 (6.79)	.0134 (4.29)	.0190 (6.98)
Feb 17-Mar 1	.0260 (10.48)	.0227 (7.31)	.0279 (9.82)
March 3–15	.0321 (12.83)	.0303 (9.92)	.0331 (11.46)
March 17–29	.0429 (16.13)	.0436 (12.54)	.0425 (14.01)
April 14–26	.0614 (21.66)	.0608 (17.21)	.0617 (19.33)
April 28-May 10	.0608 (20.30)	.0635 (16.33)	.0588 (18.45)
May 12–24	.0633 (21.85)	.0651 (17.53)	.0621 (18.83)
May 26-June 7	.0675 (22.23)	.0687 (17.50)	.0665 (19.31)
June 9–21	.0713 (23.99)	.0717 (18.18)	.0708 (21.59)
June 23-July 5	.0695 (22.45)	.0683 (17.85)	.0702 (19.76)
July 21-Aug 2	.0766 (25.64)	.0756 (18.98)	.0772 (24.04)
Aug 4–16	.0768 (26.97)	.0765 (20.28)	.0769 (23.79)
Aug 18–30	.0870 (31.42)	.0874 (23.61)	.0866 (27.82)
Sept 1–13	.0920 (31.98)	.0904 (23.67)	.0929 (28.83)
Sept 15–27	.0986 (31.67)	.0966 (22.94)	.0998 (29.47)
Sept 29-Oct 11	.1047 (35.31)	.1031 (27.11)	.1056 (31.24)
Dec 1–13	.1105 (33.19)	.1038 (26.72)	.1151 (29.32)
2022			
Dec 29-Jan 10	.1386 (31.72)	.1232 (22.70)	.1489 (31.85)
Jan 26-Feb 7	.1978 (54.17)	.1777 (41.43)	.2112 (52.93)
March 2–4	.2076 (58.87)	.1922 (43.40)	.2177 (57.98)
March 30-April 11	.2071 (50.94)	.1951 (41.03)	.2152 (47.46)
Constant	.2691	.2408	.2788
Adjusted R^2^	.0543	.0471	.0586
N	1,578,69	640,588	938,103

Notes: excluded reference categories: not working; white Hispanic; January 6^th^-18^th^. Equations include a full set of state fixed effects. T-statistics in parentheses.

The young again have the highest incidence of COVID-19, presumably in part because they have been vaccinated the least as do white Hispanics; women, those who are separated, and the least educated—especially high school dropouts. A recent paper [32], reported that the young in the UK are also at greatest risk of adverse mental health outcomes. Workers—especially in the public sector—had higher COVID-19 diagnosis rates than non-workers consistent with the possibility that being at work–or commuting to it–raised the possibility of infection. As expected, the time dummy coefficients rise over time with large jumps in 2022 as Omicron arrived.

## 5. Seasonality

One issue worth addressing is the extent to which our results are driven by seasonality given that the literature suggests mental health worsens in wintertime and improves in the summer months when there is more daylight. In a comprehensive recent paper [33] looked at insurance claims data for over 150 million US citizens (2003 and 2014) and virtually all Swedes (1980–2013) relating to psychiatric disorders. They concluded "*it appears that psychiatric disorders follow a strong seasonal prevalence variation*, *closely resembling that previously described for unipolar depression*. *The most probable explanation for this observed seasonality*, *we believe*, *involves cyclic changes of exposure to solar light*, *which*, *in turn*, *affects circadian clock rhythms*.*"*

This is confirmed in internet search data. Seasonality in Google Trend searches in Ontario, Canada for the period 2012–2017, has also been examined [34] in relation to terms including ’anxiety’, ’depression’ and general health terms. They found peak search volumes over the winter months and troughs during the summer months. An earlier study [35] examined seasonality in searches for the word ’depression’ using the search engine database, Google Insights for Search, of trends from January 1^st^ 2004 to June 30, 2009. They found the trends were different in the northern and southern hemispheres, but both peaked in winter. An examination of nearly half-a-billion Twitter and Facebook social media posts found that cold weather, in particular, was associated with an increase and a decrease in negative and positive expressions of sentiment, respectively [36]. They speculated that a winter decrease in outdoor activity may leave more time for internet searches.

As a check on the extent of seasonality we examined the 2012–2020 Behavioral Risk Factor Surveillance System (BRFSS) data collected by the Center for Disease Control (CDC), public use data files (https://www.cdc.gov/brfss/) and checked on the monthly variation in their main measures of unhappiness–the number of bad mental health days and the rate of despair. The question is "*Now thinking about your mental health*, *which includes stress*, *depression*, *and problems with emotions*, *for how many days during the past 30 days was your mental health not good*?" There are 12,000 observations in the 2020 survey for 2021.

We derive a binary variable set to one if every day of the last thirty was a bad mental health day. It is coded zero otherwise. In total there are around 4,006,037 observations on both. Bad mental health days is coded from zero through thirty with a weighted mean of 3.92 while despair has a mean of 5.85%. In column 1 we report the mean weighted data by interview month for bad mental health days and in column 2 for despair. In both cases, December is the peak unhappiness month. Interestingly, and contrary to the earlier papers, we find the lowest rates of unhappiness on both measures are in March.

**Table:** 

	Bad mental health days	Despair
January	3.81	5.77
February	3.82	5.77
March	3.76	5.50
April	3.90	5.87
May	4.02	6.00
June	3.96	5.88
July	3.85	5.71
August	3.94	5.81
September	4.00	5.88
October	4.01	6.06
November	3.97	5.83
December	4.04	6.16
All	3.92	5.85

Of note is that the peak month for bad mental health days and despair in our BRFSS data is December, so perhaps there is a seasonal effect operating in our HPS data, as December 2021 is a high point. However, July 2020 is another high point and the fourth lowest in the BRFSS. In our view it does not seem that seasonal effects are what is driving the time path of well-being in the period 2020 to 2022. Instead, they appear to be driven in large part by the path of COVID-19, and of course the accompanying lockdowns and slowing in economic activity. Temperature may well be a driving force in the spread of COVID-19 given that when it is cold people have been forced indoors at close quarters. At the time of writing, May 6^th^, 2022 COVID-19 infections are the lowest in Alabama, Wyoming, Arkansas and Mississippi at 5 per 100000 and highest in the North East in Rhode Island (57), Maine (55), Vermont (51) and Massachusetts (45) with case rates /100000 in parentheses (https://www.nytimes.com/interactive/2021/us/COVID-19-cases.html).

## 6. Discussion and conclusion

According to [15] “*the number of people looking for help with anxiety and depression has skyrocketed*” since the beginning of the COVID-19 pandemic. Between January and September 2020 315,220 people took their ‘anxiety screen’, an increase of 93% over 2019. Over half a million (534,784) took the depression screen–a 62% increase. The percentage reporting thoughts of suicide and self-harm is also rising. They say: “*young people are struggling most with their mental health*.”

We find that mental ill-health rose in 2020 through July 2020 and then improved through the summer of 2020 before hitting another peak at the end of 2020. Mental health then improved through July 2021 before unhappiness picked up again through September 2021 and has stayed roughly constant since. Mental health was better at week 44 than it was in week 1 of the HPS.

The highest probability of being diagnosed with COVID-19 is among those ages 20–24. During this period anxiety, depression and worry are also highest age 20–24 and then decline with age, something that we think is specific to this COVID-19 era. This contrasts with the usual finding that unhappiness is hump-shaped in age [37–39]. We did find though that being prescribed with a prescription drug for depression followed the tradition hump shaped path in age.

We find that COVID-19 inflow rates in the state*year cell impact mental health. As COVID-19 cases rose from March 2020 mental health deteriorated, controlling for whether the individual had ever personally had COVID-19. As cases peaked and troughed well-being followed. Of note though is that our measures of unhappiness did not peak to anywhere near the extent to which cases did at the start of 2022, so there appears to have been some adaptation, perhaps due to the lower incidence of death and vaccine roll-out. The coefficient on our COVID-19 inflow variable was highest in 2020 and then declined over time.

We find that the COVID-19 inflow rate variable had an especially large negative impact on the well-being of women, who saw a relatively larger improvement in wellbeing in the second half of 2021 and subsequently than men. We showed that having been vaccinated improved mental health for women no matter what their level of education. College educated men also had better mental health if they had been vaccinated, but the opposite was true for less educated men, suggesting the possibility that being vaccinated for them was a social stigma.
